# The impact of health shocks on poverty vulnerability: evidence from rural households in China

**DOI:** 10.3389/fpubh.2025.1657269

**Published:** 2025-08-29

**Authors:** Xiaonan Zhao, Xinzheng Wang, Zhou Ren, Cancan Zhang

**Affiliations:** School of Civil Engineering and Architecture, Nanyang Normal University, Nanyang, China

**Keywords:** health shocks, poverty vulnerability, agricultural production income, wage income, rural households

## Abstract

**Background:**

“Poverty due to illness” presents the core challenge for both the Healthy China Initiative and the consolidation of poverty alleviation achievements, concerning health equity and sustainable social development. Existing studies have predominantly assessed the impact of health shocks on poverty from a static perspective. However, they often overlook the dynamic nature of poverty, failing to account for the impact of health shocks on future poverty risks.

**Methods:**

Based on the data of the China Family Panel Studies (CFPS) from 2012 to 2020, this paper measures the poverty vulnerability under the criteria of absolute poverty and relative poverty, and empirically analyzes the impact of health shocks on rural households’ poverty vulnerability.

**Results:**

The results show that health shocks have a significant positive effect on household poverty vulnerability, and the effect is greater under absolute poverty criteria. Further analysis revealed that a higher level of education significantly mitigated the impact of health shocks on poverty vulnerability; Further analysis revealed that a higher level of education significantly mitigated the impact of health shocks on poverty vulnerability; compared to the eastern region, the exacerbating effect of health shocks on poverty vulnerability was more severe in the central and western regions, presenting a gradient difference of “eastern region < central region < western region.” In addition, we find that declines in agricultural production income and wage income are important channels through which health shocks affect poverty vulnerability.

**Conclusion:**

This study provides the policy basis for the government to establish the “pre-warning” mechanism of poverty and take effective measures to prevent the large-scale return to poverty.

## 1 Introduction

Poverty poses a formidable challenge to the development of human society. For a long time, countries around the world have made great efforts to eradicate poverty and have achieved certain results. Nevertheless, the shock of uncertainty risk poses a significant obstacle to the objective of poverty reduction. In 2020, approximately 70 million individuals worldwide fell back into extreme poverty due to the impact of the COVID-19 pandemic, marking the highest recorded number since the initiation of poverty monitoring in 1990 (1). China has made notable achievements in achieving its poverty reduction targets. However, mounting risks and uncertainties, both domestically and internationally, have exacerbated the challenges associated with establishing a long-term mechanism for poverty reduction. Problems such as health and aging reduce the risk coping capacity of rural households, putting them at risk of returning to poverty (2). Realizing stable poverty eradication among marginalized groups is not only an important criterion for evaluating the success of poverty eradication but also a prerequisite for promoting common prosperity. Hence, preventing and mitigating poverty risks faced by rural households and strengthening their risk resilience are essential to achieving sustainable poverty reduction.

As the basic unit of risk management, the household is both risk-taker and risk-resistant. Various sudden risk shocks not only test the family’s risk management and coping ability, but also weaken the family’s risk resistance ability. Lack of internal power, difficulty in sustainable income increase and inadequate social security are common problems in rural areas, particularly the vulnerable groups’ limited resilience against diverse risks. This is also the root cause of rural families falling into poverty. Extreme weather, drought, epidemics and health shocks are among the risks closely associated with rural poverty (3, 4). These risks may trigger a chain reaction in economic and social aspects. The lack of medical resources, especially in rural areas, increases the risk of “poverty due to illness.” Health is a prerequisite for human production and life, serving as a safeguard for families against the shocks of various risks (5). If the health problems of farmers cannot be effectively addressed, it will affect the livelihood activities of families. Especially for families that have escaped poverty, once they encounter the impact of health risks, the family will face the risk of falling back into poverty. Therefore, improving the health security of households is crucial for poverty alleviation, livelihood sustainability and rural development.

Vulnerability to poverty reflects the future well-being of households and the associated risks (6, 7). Health shocks are one of the important factors affecting the level of household welfare (8). However, the causal relationship between health and poverty remains controversial. On the one hand, health affects poverty by influencing physical and human capital. There is growing evidence that health shocks affect poverty primarily in terms of labor supply, labor productivity, employment, household income and consumption (4, 9, 10). For instance, Labor participation can improve the capacity of households to cope with risks, which has a mitigating effect on poverty vulnerability. Poor health reduces individual labor participation, which lead to a decrease in household income and an increase in the risk of poverty (11). Further research finds that the negative impact of health shocks on household income varies significantly across income groups (12). Moreover, the decline in labor productivity and working hours caused by health shocks is detrimental to the accumulation of household wealth (13). On the other hand, some scholars have pointed out that income inequality and poverty can also affect health status in turn. The study found that poverty has a negative impact on an individual’s physical and mental health, especially for older people in rural areas who lack resilience to risks (14). In addition, energy poverty can also harm health, thereby trapping residents in poverty traps (15).

With the overall victory in poverty alleviation, the focus of China’s poverty management has gradually shifted to preventing the large-scale return to poverty. However, the measurement of poverty in existing studies is static, mainly reflected as an ex-post response to welfare loss, and may not serve as an effective tool to identify the risk of returning to poverty (16). The impact of disease risk on poverty is not temporary, but can also have long-term effects. This is largely due to the fact that health shocks can reduce an individual’s ability to earn income and thrive, leading to households falling into chronic poverty (17, 18). Thus, a static assessment based on poverty cannot reveal the long-term effects of health risks. Poverty vulnerability is a forward-looking measure of poverty, reflecting the probability of a household or individual falling into poverty due to an uncertain risk shock (19, 20). This indicator makes it possible to identify groups at risk of returning to poverty in a timely manner and to develop targeted measures to prevent them from falling into poverty in the future. At the same time, health poverty alleviation, as an important part of the targeted poverty alleviation strategy, plays a positive role in preventing the return to poverty due to illness. However, China’s impoverished population is overly dependent on government subsidies and have yet to make the transition from “blood transfusion” to “blood creation” (21, 22). Moreover, rural residents have just emerged from poverty and their ability to withstand risks is still limited. Therefore, the problem of returning to poverty faced by rural families cannot be ignored. Accurately identifying the relationship between health and poverty vulnerability and enhancing the resilience of rural residents to health risks can provide an effective guarantee for the comprehensive victory of poverty alleviation. Therefore, this paper establishes an analytical framework under poverty vulnerability, re-examines the issue of health and poverty, and provides reference for achieving the goal of sustainable poverty reduction.

To fill the gaps in existing research, this paper uses the CFPS data to conduct an empirical study on the impact of health shocks on poverty vulnerability. Its possible marginal contribution lies in: (1) Within the framework of sustainable livelihoods, it is revealed how health shocks, as a key risk, can undermine farmers’ income, triggering a dynamic “health-poverty trap,” thereby elevating the analysis from static consequences to the level of pre-event risks. (2) Conduct a rigorous empirical investigation of the relationship between health shocks and poverty vulnerability within the specific and significant context of rural areas in China. China’s rural areas have undergone rapid social and economic changes and medical security reforms (such as the New Rural Cooperative Medical Scheme and the integration of urban and rural medical insurance), and the mechanisms and consequences of health shocks may be unique in this context. (3) Identify the characteristics of the groups that are most vulnerable to health shocks and are prone to falling into poverty, so as to provide a basis for designing more targeted health poverty alleviation and social protection policies.

The remainder of the paper is organized as follows: section 2 presents the theoretical analysis and research hypotheses. Section 3 describes the methodology, variable and data sources. Section 4 introduces the results of the analysis. Section 5 provides a discussion of the results. Section 6 summarizes the conclusions and corresponding policy recommendations.

## 2 Theoretical analysis and research hypothesis

### 2.1 Health shocks and poverty vulnerability

As major participants in the rural market economy, rural households are exposed to the possibility of risky shocks in both production and life. Poor resilience to risks, a single livelihood structure, and an unstable source of income may push farmers into poverty (23, 24). As an important part of human capital, health is a key factor affecting the income stability and risk resistance of rural residents. In general, health risk refers to the loss of welfare caused by a family member being threatened with illness over a period of time. The greater the health risks that farmers face, the greater the likelihood that families will fall into poverty in the future.

The impact of health risks on the poverty vulnerability of rural households is mainly reflected in the following three aspects. First, health shocks can lead to higher medical costs, which increase the financial burden on families (25). This is especially true for rural households, which have limited access to and ability to obtain income. Poverty can occur when health risks are beyond a family’s ability to cope. Second, health shocks reduce households’ human capital, which is detrimental to income-generating capacity (26). Limited sources of income and unstable income are common phenomena in rural areas. An increase in family spending on health care inevitably leads to a decrease in spending on education (27). Compared with “poverty due to illness,” “poverty due to learning” is also an important factor that causes rural families to fall into poverty (28). However, the income risk from health shocks is smaller for households with higher levels of education (29). Third, emotional problems triggered by health shocks may cause cognitive biases among family members, thus affecting major family decisions. Faced with risk shocks, the decision-making behavior of farmers will have a significant impact on the quality of family poverty alleviation (30). In particular, the risk decision-making behaviors of impoverished households may impair their ability to improve family welfare and manage risk in the future, thereby increasing their poverty risk (31, 32). Based on the above analysis, we propose hypothesis 1:

*Hypothesis 1*. Health shocks increase the vulnerability of rural households to poverty.

### 2.2 Health shocks, income effects and poverty vulnerability

One important theoretical perspective for understanding the relationship between health shocks and poverty vulnerability is the theory of capability poverty. This theoretical paradigm shifts the focus of poverty analysis from merely the lack of income (a static perspective) to the analysis of the deprivation of capabilities. This theory emphasizes the significance of health as a core capability. Health shocks directly undermine an individual’s ability to resist risks. Good health is of vital importance for the ability to work effectively, earn income and accumulate assets. Poor health directly impairs physical and cognitive functions, reducing labor participation, productivity and income potential (11). Studies have shown that being ill or caring for patients leads to labor loss (reduced working hours and decreased productivity), which directly affects family income (33). Some scholars have also pointed out that the fragmentation of social support may also affect informal security (such as support from relatives and friends) (34).

Income is the main source and important reason for rural residents to fall into poverty (35). Low-income groups, in particular, have high livelihood vulnerability and are highly exposed to external shocks that lead them into poverty (36). Good health is the guarantee that an individual can earn an income. Individuals experiencing health shocks are usually in an unhealthy state, which inevitably affects labor efficiency (37). The most direct manifestation of this effect is the decline in household labor income. From the perspective of income composition, household income includes wage income, business income, property income, transfer income and other income (38, 39). For rural households, agricultural production income and wage income are closely related to their poverty vulnerability.

According to the sustainable livelihood theory, families will adjust their livelihood strategies based on their livelihood capital to achieve sustainable development. The choice of livelihood strategies may lead to the changes of household income structure (40). For families engaged in agricultural operations, income from agricultural production is the main source of family income. However, the fragmented management of land in China is not enough to support large-scale mechanized production (41). As a result, existing agricultural labor is mainly physical activity, making household income more dependent on an individual’s physical condition. As a result, health shocks will inevitably lead to a reduction in agricultural labor hours and a decrease in agricultural productivity, which is not conducive to the accumulation of agricultural production income (42). For households engaged in non-agricultural operations, wage income is an important part of the family income source. When farmers with non-farm employment experience a health shock, they spend less time working outside the home. Additionally, the health condition of workers diminishes their ability and productivity, potentially leading to a reduction in their wage earnings (43). In the long run, health shocks can also reduce the quality of the household labor force, which is detrimental to the accumulation of household wealth (44). Therefore, we propose hypothesis 2:

*Hypothesis 2*. Health shocks can reduce income from agricultural production and wage income, leading to an increase in household poverty vulnerability.

## 3 Materials and methods

### 3.1 Methodology

#### 3.1.1 Poverty vulnerability measurement

The World Bank defines poverty vulnerability as the likelihood that an individual or family will experience a decline in their welfare level below the poverty threshold due to a risk shock (45). Subsequent studies have further expanded upon the two key elements of risk and welfare as defined by the World Bank, and have mainly developed three methods for calculation. The Vulnerability as Uninsure Exposure to Risk (VER) posits that poverty vulnerability refers to the sensitivity of an individual’s or family’s welfare level to risk shocks (46). The Vulnerability as Low Expected Utility (VEU) posits that poverty vulnerability refers to a situation where, under conditions of uncertainty, the welfare effects brought about by an individual or family’s expectations of consumption are lower than the welfare effects stipulated by the poverty line standard (47). And the Vulnerability as Expected Poverty (VEP) refers to the likelihood that an individual or family will have a future welfare level lower than the poverty threshold (48). The VEP method not only takes into account the current characteristics of individuals or families, but also includes their future welfare conditions, and thus has a forward-looking feature (49, 50). In contrast, VEU and VER also emphasize the impact of risk shocks and their effects. Risk response is only analyzed as an influencing factor for poverty vulnerability, and their measurement results cannot identify the risk of people falling into poverty in the future. The VEP method, however, focuses more on the welfare consequences resulting from poverty vulnerability-namely, the risk of falling into poverty in the future. Therefore, in this paper, the VEP method, which pays more attention to the possibility of families falling into poverty in the future, is adopted to measure poverty vulnerability. The steps for calculating poverty vulnerability are as follows:

In the first step, the per capita consumption equation is estimated and the OLS estimation is continued after obtaining the residual term:


$$\ln Y_{i}={\mathrm{\alpha X}}_{i}+e_{i}$$

$${\hat{e}}_{i}^{2}={\mathrm{\beta x}}_{i}+\varepsilon_{i}$$

Where, $Y_{i}$ denotes the annual per capita consumption of the ith household. $X_{i}$ represents the variables of head of household characteristics and household characteristics that affect the annual consumption per capita of the ith household. Among them, the characteristics of the household head include age, gender, education level, etc. Family characteristics include family size, labor force ratio, land assets, family income, etc. $e_{i}$ is the residual term, representing the fluctuation term of household consumption. $\varepsilon_{i}$ is the random error term.

In the second step, the weights are constructed based on the first step, and then the consumption means and residual squares are weighted in the regression. Where is a consistent estimate of the consumption expectation and is a consistent estimate of the consumption variance:


$$\hat{\mu }=\hat{E\;}[\ln Y_{i}\;|\;X_{i}]=X_{i}{\hat{\alpha }}_{\text{FGLS}}$$

$$\hat{\sigma_{i}^{2}}=\hat{\mathrm{Var}}\;[\ln Y_{i}\;|\;X_{i}]=X_{i}{\hat{\beta }}_{\text{FGLS}}$$

In the third step, the poverty line is selected to calculate the poverty vulnerability of the household:


$$\hat{{\mathrm{VUL}}_{i}}=\hat{\mathrm{Pr}}({\mathrm{lnY}}_{i}\leq \text{lnPoor})=\Phi [\frac{\text{lnPoor}-X_{i}{\hat{\alpha }}_{\text{FGLS}}}{\sqrt{X_{i}{\hat{\beta }}_{\text{FGLS}}}}]$$

#### 3.1.2 Model construction

In order to analyze the impact of health shocks on household poverty vulnerability, this paper constructs the following model:


$$\mathrm{Vu}l_{\mathrm{it}}=\alpha +\text{βHealthshoc}k_{\mathrm{it}}+\gamma X_{\mathrm{it}}+\mu_{i}+\lambda_{t}+\varepsilon_{\mathrm{it}}$$

Where $\mathrm{Vu}l_{\mathrm{it}}$ represents the poverty vulnerability of the i household in the year t . $\text{Healthshoc}k_{\mathrm{it}}$ represents the health shock of the i household in the year t , and the values are 0 and 1. $X_{\mathrm{it}}$ is a set of control variables such as age, gender, marriage and education level for household head characteristics, and household size, labor force share, land assets and housing for household characteristics; $\mu_{i}$ denotes household fixed effects; $\lambda_{t}$ denotes year fixed effects; $\varepsilon_{\mathrm{it}}$ denotes a random error term.

### 3.2 Variables

#### 3.2.1 Dependent variable

Regarding the measurement of poverty vulnerability, existing studies prefer to identify poverty vulnerability through consumption indicators. Compared with income, consumption can better reflect the level of household welfare, and the error of consumption data is small (32, 51). Therefore, this paper uses the consumption criterion (annual consumption per capita) to measure the poverty vulnerability of households. As for the poverty line, the degree of poverty varies among households. Therefore, this paper assesses poverty vulnerability using two criteria: absolute poverty and relative poverty. With regard to the absolute poverty line, the poverty standard set by the World Bank is $1.90 per person per day and $3.2 per person per day, of which $3.2 per person per day is the international poverty line for low- and middle-income countries (52, 53). Therefore, this paper takes the standard of $3.2 per person per day as the baseline poverty line, and adopts the standard of $1.9 per person per day for robustness test. In our analysis, we determined the corresponding poverty thresholds based on the Purchasing Power Parity (PPP) and the Consumer Price Index (CPI) indices. For the relative poverty line, we use 50% of the median annual income per capita as the baseline relative poverty line, and 60% of the median annual income per capita as a robustness test (54, 55).

#### 3.2.2 Independent variable

A health shock refers to a situation where one is severely affected by an illness within a certain period of time. According to the standards set by the World Health Organization (WHO), catastrophic medical expenses are defined as medical expenditures accounting for 40% or more of a household’s non-food consumption expenses (27). This study focuses on identifying high-risk families that are prone to falling into poverty due to illness. Considering that the rural income level in China is relatively low, even though the medical expenses may not be very large in absolute terms, they may still exceed the affordability limit, thus failing to meet basic consumption needs and falling into poverty. Therefore, based on the above definition, this article defines health shocks as medical expenditures accounting for 10% or more of household income. This approach helps to avoid underestimating the occurrence of serious illnesses among low-income individuals to a certain extent. It is worth noting that for many impoverished families, there may be cases where they need medical treatment due to illness but are unable to receive it because of their poverty. This article only focuses on families that have actually incurred medical expenses, and it neglects those poor families that cannot afford medical treatment. This might still lead to an underestimation of the impact of health shocks on vulnerable families.

#### 3.2.3 Control variables

In order to exclude the influence of other factors on poverty vulnerability as much as possible, we controlled for factors such as individual and household characteristics in the model. Drawing on previous studies, we selected the characteristics of the household head and family as the control variables (3, 56). Among them, household head characteristics variables include age, gender, marriage and education level. Household characteristics variables include dependency ratio, labor force ratio, housing, land and savings.

### 3.3 Data source

The data used in this paper comes from the China Family Panel Studies (CFPS) conducted by Peking University. The CFPS data underwent seven surveys, covering 25 provinces, autonomous regions and municipalities across the country, with good representation. At the same time, the data covers the information of individuals, families and communities at three levels, which provides conditions for the research of this paper. Before the empirical analysis, we processed the data: First, we delete the 2010 sample due to the lack of core explanatory variables; Second, this paper excludes the urban sample and retains only the rural sample; Third, to minimize the influence of the epidemic and enhance the universality of the conclusions, we extracted samples that were tracked in 2012, 2014, 2016, 2018, and 2020; Finally, the samples with missing variable information are deleted. After the above processing, we obtained 9,285 samples from 1857 rural households. Descriptive statistics of variables are shown in Table 1.

**Table 1:** Variable description and descriptive statistics.

Variables	Definitions	Obs	Mean	SD	Min	Max
Dependent variable						
Poverty vulnerability	Absolute poverty line: $3.2 per day	9,285	0.1178	0.1814	0	1
	Relative poverty line: 50% of median per capita household income	9,285	0.1261	0.1873	0	1
Independent variable						
Health shock	If the family has experienced a health shock, the value is 1; Otherwise 0	9,285	0.3304	0.4704	0	1
Control variables						
Age	Age of head of household	9,285	51.6471	11.9812	17	87
Gender	Gender of head of household (Male = 1; Female = 0)	9,285	0.5776	0.4940	0	1
Marriage	Marital status of head of household (Married = 1; Unmarried = 0)	9,285	0.9172	0.2756	0	1
Education	Educational level of the head of household	9,285	2.2619	1.0445	1	5
Dependency ratio	Proportion of minors and older persons in households	9,285	0.1471	0.2604	0	1
Labor	Proportion of household labor force	9,285	0.6507	0.2866	0	1
House	Whether the family owns a home (Yes = 1; No = 0)	9,285	0.9575	0.2018	0	1
Land	Logarithm of land assets	9,285	8.7373	3.6690	0	14.9579
Savings	Logarithm of household savings	9,285	6.3408	4.5647	0	15.3196
Social insurance	Whether the family participates in social insurance (Yes = 1; No = 0)	9,285	0.8883	0.3150	0	1
Subsidy	Whether the family receives government subsidies (Yes = 1; No = 0)	9,285	0.5475	4,978	0	1

## 4 Results

### 4.1 Baseline regression results

Table 2 shows the impact of health shocks on poverty vulnerability. Columns 1 and 4 report the regression results without adding any control variables. We can find that the coefficients of health shock are 0.0870 and 0.0754, respectively. Columns 2 and 5 report the regression results for controlling household head characteristics, showing that the coefficients of health shocks are 0.0852 and 0.0733, respectively. Columns 3 and 6 show regression results that further control for family characteristic variables and find that the coefficients of health shocks are 0.0834 and 0.0707, respectively. It can be found that the estimated coefficients of health shocks are positive for both absolute and relative poverty criteria and pass the significance test at the 1% level. The results show that health shocks significantly increase the poverty vulnerability of households, and the impact of health shocks on poverty vulnerability is greater under absolute poverty criteria. Therefore, hypothesis 1 is verified.

**Table 2:** Estimation results from benchmark regressions.

Variables	(1)	(2)	(3)	(4)	(5)	(6)
	Absolute poverty line			Relative poverty line		
Health shock	0.0870^***^	0.0852^***^	0.0835^***^	0.0754^***^	0.0733^***^	0.0708^***^
	(0.0047)	(0.0046)	(0.0045)	(0.0046)	(0.0044)	(0.0043)
Age		0.0036^***^	0.0037^***^		0.0036^***^	0.0036^***^
		(0.0003)	(0.0003)		(0.0003)	(0.0003)
Gender		0.0090^**^	0.0096^**^		0.0050	0.0058
		(0.0044)	(0.0043)		(0.0043)	(0.0043)
Marriage		−0.0131	−0.0138		−0.0108	−0.0114
		(0.0010)	(0.0098)		(0.0092)	(0.0091)
Education		−0.0230^***^	−0.0241^***^		−0.0253^***^	−0.0263^***^
		(0.0031)	(0.0030)		(0.0030)	(0.0030)
Dependency ratio			−0.1370^***^			−0.0755^***^
			(0.0133)			(0.0140)
Labor			−0.1380^***^			−0.1380^***^
			(0.0100)			(0.0104)
House			0.0025			−0.0110
			(0.0083)			(0.0084)
Land			0.0006			0.0014^**^
			(0.0006)			(0.0006)
Savings			−0.0013^***^			−0.0015^***^
			(0.0004)			(0.0004)
Social insurance			−0.0136^***^			−0.0194^***^
			(0.0061)			(0.0064)
Subsidy			−0.0158^***^			−0.0106^**^
			(0.0041)			(0.0042)
Time-FE	Yes	Yes	Yes	Yes	Yes	Yes
Household-FE	Yes	Yes	Yes	Yes	Yes	Yes
Observations	9,285	9,285	9,285	9,285	9,285	9,285
*R* ^2^	0.5831	0.6058	0.6215	0.6001	0.6223	0.6366

Robust standard errors in parentheses. ***p < 0.01, **p < 0.05.

### 4.2 Robustness tests

#### 4.2.1 Replacement of poverty standards

In the baseline regression, we mainly used $3.20 per person and 50% of median household income per capita as the poverty line. In this section, we conduct a robustness test using $1.90 per person and 60% of median household income per capita as the poverty threshold. The results are shown in columns 1 and 2 of Table 3. Regardless of the poverty line used, the coefficients of health shocks are significantly positive at the 1% level. This provides evidence that health shocks increase households’ vulnerability to poverty and suggests that the results of this study are robust.

**Table 3:** Robustness test results: change the measurement of the dependent variable.

Variables	(1)	(2)	(3)	(4)
	Change poverty standard		Transform poverty measurement	
	$1.9 per day	60% of the median	$3.2 per day	50% of the median
Health shock	0.0277^***^	0.0997^***^	0.0662^***^	0.0632^***^
	(0.0025)	(0.0048)	(0.0064)	(0.0072)
Control variables	Yes	Yes	Yes	Yes
Time-FE	Yes	Yes	Yes	Yes
Household-FE	Yes	Yes	Yes	Yes
Observations	9,285	9,285	9,285	9,285
R^2^	0.4414	0.7194	0.4373	0.4163

Robust standard errors in parentheses. ***p < 0.01.

#### 4.2.2 Changing the measurement of poverty vulnerability

The vulnerability of households to poverty can also be measured by “whether the household is vulnerable.” The explained variable is a binary variable. If the probability of a family falling into poverty in the future is greater than or equal to the vulnerability threshold, the family is considered to be poverty-vulnerable. Otherwise, the household is not poverty-vulnerable. Referring to the existing literature, we choose 50% as the vulnerability threshold for poverty vulnerability measurement (57, 58). The results are shown in columns 3 and 4 of Table 3. It can be seen that neither the sign nor the significance of the health shocks changed significantly, indicating that our results remain robust even after using different measures.

#### 4.2.3 Changing the measurement of the independent variable

We measure the core independent variable of the health shock by the medical expenditure exceeding 10% of the household income. However, the results may be sensitive to the chosen threshold. Therefore, we further use 5 and 15% as thresholds to conduct sensitivity checks. The results are shown in Table 4. It can be observed that, under different thresholds, the impact of the health shock on poverty vulnerability remains significantly positive at the 1% level. The result was once again confirmed to be reliable.

**Table 4:** Robustness test results: change the measurement of the independent variable.

Variables	(1)	(2)	(3)	(4)
	Absolute poverty line		Relative poverty line	
Health shock (5%)	0.0645^***^		0.0561^***^	
	(0.0036)		(0.0036)	
Health shock (15%)		0.0960^***^		0.0792^***^
		(0.0050)		(0.0048)
Control variables	Yes	Yes	Yes	Yes
Time-FE	Yes	Yes	Yes	Yes
Household-FE	Yes	Yes	Yes	Yes
Observations	9,285	9,285	9,285	9,285
*R* ^2^	0.6121	0.6263	0.6309	0.6387

Robust standard errors in parentheses. ***p < 0.01.

### 4.3 Discussion on endogeneity

There may be reverse causality between the explanatory variables and the explained variables, leading to endogeneity problems. On the one hand, low-income people lack sufficient capital and the ability to avoid risks, and poverty affects their willingness to pay when faced with health risks. On the other hand, large medical expenditures resulting from health shocks can place a heavy financial burden on families, thus increasing the risk of poverty faced by families. Therefore, in order to alleviate the endogeneity problem, this paper adopts the instrumental variable method for 2sls regression. Referring to existing studies, the mean value of “health shocks” in the sample villages was selected as the instrumental variable (59).

Table 5 reports the regression results of the instrumental variable method. Columns 1 and 3 report the results of the first-stage regressions, respectively. The estimated coefficients of the instrumental variables are all significantly positive, indicating that the correlation requirement is satisfied. Meanwhile, the value of Kleibergen-Paap rk LM statistic is 316.991, indicating that there is no under-identification of instrumental variables; The value of Cragg-Donald Wald *F*-statistic is 650.115, which is greater than the critical value of 16.38 at the 10% level of the Stock-Yogo weak instrumental variable test, indicating that there is no weak instrumental variable. Columns 2 and 4 report the results of the second-stage regressions, respectively. It can be observed that the estimated coefficient of health shocks remains significantly positive at the 1% level, indicating that health shocks have a significant positive effect on household poverty vulnerability after accounting for endogeneity. It is worth noting that our IV strategy addresses the endogeneity issue. However, the moderate goodness-of-fit in the first stage (partial *R*^2^ = 0.28) suggests that our estimated values may be biased toward the OLS results. Although considering the confirmed endogeneity, the IV estimates may still be more reliable than the OLS estimates. Therefore, we emphasize qualitative rather than precise quantitative interpretation of the IV results.

**Table 5:** Endogeneity test results.

Variables	(1)	(2)	(3)	(4)
	Absolute poverty line		Relative poverty line	
	2SLS first Stage	2SLS second Stage	2SLS first Stage	2SLS second Stage
	Health shock	Poverty vulnerability	Health shock	Poverty vulnerability
Health shock		0.1287^***^(0.0157)		0.1009^***^(0.0145)
IV	0.9149^***^(0.0409)		0.9149^***^(0.0409)	
Control variables	Yes	Yes	Yes	Yes
Household-FE	Yes	Yes	Yes	Yes
Time-FE	Yes	Yes	Yes	Yes
F statistic	48.06		48.06	
Kleibergen-Paap rk LM statistic		316.991 (*p* = 0.0000)		316.991 (*p* = 0.0000)
Cragg-Donald Wald *F* statistic		650.115		650.115
Centered *R*^2^	0.2893	0.1366	0.2893	0.1304
Observations	9,285	9,285	9,285	9,285

Robust standard errors in parentheses. ***p < 0.01.

### 4.4 Heterogeneity test

#### 4.4.1 Heterogeneity of education level

Health shocks may have heterogeneous effects on families with different levels of education. This article divides the sample families into high-education families and low-education families based on the boundary of high school graduation. Families with a high level of education are assigned a value of 1, otherwise 0. The results are shown in columns 1 and 3 of Table 6. It can be observed that the interaction coefficient between health shocks and higher education is significantly negative at the 1 and 10% levels, respectively. This indicates that a higher level of education can significantly reduce the impact of health shocks on poverty vulnerability, but the alleviation effect is stronger under the absolute poverty standard. The possible reason is that under the absolute poverty standard, education can more directly assist families in crossing the fixed poverty line by enhancing their income capacity and reducing medical expenses. Under the relatively poverty standard, as the poverty line rises dynamically along with the overall income level and given that health shocks may exacerbate multiple disadvantages, the individual income improvement brought about by education may not fully offset the effects of social inequality or the increase in the threshold, thus the alleviation effect is relatively weak. This disparity highlights the need for educational poverty alleviation policies to be combined with poverty measurement criteria and structural interventions (such as medical security systems, social security, etc.) in order to enhance their effectiveness.

**Table 6:** Heterogeneity analysis results.

Variables	(1)	(2)	(3)	(4)
	Absolute poverty line		Relative poverty line	
Health shock	0.0847^***^	0.151^***^	0.0720^***^	0.0570^***^
	(0.0045)	(0.0120)	(0.0044)	(0.0071)
Health shock* Highly educated	−0.0526^***^		−0.0414^*^	
	(0.0189)		(0.0230)	
Health shock* Central region		0.0223^**^		0.0197^*^
		(0.0111)		(0.0108)
Health shock* Western region		0.0287^***^		0.0205^**^
		(0.0105)		(0.0102)
Control variables	Yes	Yes	Yes	Yes
Time-FE	Yes	Yes	Yes	Yes
Household-FE	Yes	Yes	Yes	Yes
Observations	9,285	9,285	9,285	9,285
*R* ^2^	0.6181	0.6369	0.6329	0.6369

Robust standard errors in parentheses. ***p < 0.01, **p < 0.05, *p < 0.1.

#### 4.4.2 Regional heterogeneity

The impact of health shocks on poverty vulnerability may show regional differences among households. We analyzed regional heterogeneity by constructing interaction terms between health shocks and the central region, and the western region (with the eastern region as the reference group). The results are shown in columns 2 and 4 of Table 6. It can be observed that the coefficients of the interaction terms are all significantly positive. This indicates that compared to the eastern region, the impact of the health shock on the exacerbation of poverty vulnerability is more severe in the central and western regions, presenting a gradient difference of “eastern region < central region < western region.” Moreover, the impact of the health shock is more pronounced for rural families in the central and western regions when measured against the absolute poverty standard. This might be because a sudden increase in medical expenses and a sharp decline in income could directly push families below the poverty line. The relative poverty standard measures the relative economic status of a family within a region, and its change is not only determined by its own income fluctuations but also influenced by the overall income distribution. Due to the relatively low overall income levels in the central and western regions and their concentrated distribution, the impact of health shocks on the relative economic status of families will be partially mitigated by the overall economic conditions of the region. Meanwhile, the existing poverty alleviation policies (such as targeted poverty alleviation and major illness insurance) can effectively alleviate the decline in absolute income, but have limited effect on improving the relative position of families in the income distribution. Moreover, the human capital loss caused by health shocks (such as the decline in labor quality and the interruption of children’s education) is lagging, resulting in a less obvious exacerbating effect on relative poverty compared to absolute poverty.

### 4.5 Mechanism analysis

In the theoretical analysis part, health shocks can influence poverty vulnerability through the income effect of labor. Therefore, to explore the potential mechanisms by which health shocks affect poverty vulnerability, we introduce two new variables, namely wage income and agricultural income. The results are shown in Tables 7, 8. It can be seen from Table 7 that health shocks have a significant positive impact on poverty vulnerability under both absolute and relative poverty standards. After we add wage income into the model, the estimated coefficients of health shocks are still significantly positive, but their sizes are reduced, suggesting that wage income plays a partial mediating effect. To ensure the robustness of the results, we further conducted a sobel test for mediating effects. The sobel test results show that the *z*-values are all significant at the 1% level, further confirming the existence of the mediating effect. Further mediation effect Bootstrap test (with 5,000 repeated sampling) revealed that the 95% confidence interval of the indirect effect did not include 0, which also indicated that the mediation effect was significant. Table 8 shows the test results of agricultural income, and we obtain similar conclusions as in Table 7. In summary, this paper confirms that wage income and agricultural income are potential mechanisms through which health shocks affect household poverty vulnerability.

**Table 7:** Mechanism test results: wage income.

Variables	(1)	(2)	(3)	(4)	(5)	(6)
	Absolute poverty line			Relative poverty line		
	Poverty vulnerability	Wage income	Poverty vulnerability	Poverty vulnerability	Wage income	Poverty vulnerability
Health shock	0.0835^***^	−1.8900^***^	0.0648^***^	0.0708^***^	−1.8900^***^	0.0547^***^
	(0.0035)	(0.0979)	(0.0035)	(0.0036)	(0.0979)	(0.0036)
Wage income			−0.0099^***^			−0.0085^***^
			(0.0004)			(0.0004)
Control variables	Yes	Yes	Yes	Yes	Yes	Yes
Time-FE	Yes	Yes	Yes	Yes	Yes	Yes
Household-FE	Yes	Yes	Yes	Yes	Yes	Yes
Observations	9,285	9,285	9,285	9,285	9,285	9,285
*R* ^2^	0.6215	0.5644	0.6499	0.6366	0.5644	0.6564
Sobel Test	0.0187^***^			0.0161^***^		
	(0.0012)			(0.0011)		
Goodman-1 (Aroian)	0.0187^***^			0.0161^***^		
	(0.0012)			(0.0011)		
Goodman-2	0.0187^***^			0.0161^***^		
	(0.0012)			(0.0011)		
Bootstrap test	[95% Conf. Interval] (0.0157224, 0.0217311)			[95% Conf. Interval] (0.0133419, 0.0189308)		

Robust standard errors in parentheses. ***p < 0.01.

**Table 8:** Mechanism test results: agricultural income.

Variables	(1)	(2)	(3)	(4)	(5)	(6)
	Absolute poverty line			Relative poverty line		
	Poverty vulnerability	Agricultural income	Poverty vulnerability	Poverty vulnerability	Agricultural income	Poverty vulnerability
Health shock	0.0835^***^	−0.6131^***^	0.0800^***^	0.0708^***^	−0.6131^***^	0.0674^***^
	(0.0035)	(0.0857)	(0.0035)	(0.0036)	(0.0857)	(0.0036)
Agricultural income			−0.0058^***^			−0.0056^***^
			(0.0005)			(0.0005)
Control variables	Yes	Yes	Yes	Yes	Yes	Yes
Time-FE	Yes	Yes	Yes	Yes	Yes	Yes
Household-FE	Yes	Yes	Yes	Yes	Yes	Yes
Observations	9,285	9,285	9,285	9,285	9,285	9,285
*R* ^2^	0.6215	0.5852	0.6289	0.6366	0.5852	0.6431
Sobel Test	0.0036^***^			0.0034^***^		
	(0.0006)			(0.0006)		
Goodman-1 (Aroian)	0.0036^***^			0.0034^***^		
	(0.0006)			(0.0006)		
Goodman-2	0.0036^***^			0.0034^***^		
	(0.0006)			(0.0006)		
Bootstrap test	[95% Conf. Interval] (0.0022096, 0.0049067)			[95% Conf. Interval] (0.0021148, 0.0047267)		

Robust standard errors in parentheses. ***p < 0.01.

## 5 Discussion

This study assesses the impact of health shocks on poverty using poverty vulnerability indicators. Our results suggest that health shocks have a significant positive effect on the poverty vulnerability of rural households. This is consistent with Ouadika’s findings (50). After controlling for endogeneity and eliminating the influence of unobservable variables, the conclusion is still reliable. The economic burden of disease and health damage are the most important factors contributing to rural poverty in China. The direct impact of health shocks is to increase the family’s medical expenditure, putting them under severe economic pressure (25). At the same time, labor time lost by family members caring for individuals in poor health further negatively affects the welfare level of the family. Therefore, the Chinese government has also adopted a series of health poverty alleviation policies, such as new rural cooperative medical care and resident medical insurance, to ward off health shocks. These measures can reduce individual health care consumption and help households reduce the risk of falling back into poverty (60). Therefore, health poverty alleviation plays an important role in poverty management in rural areas.

From the perspective of sustainable livelihoods, Labor resources, social interaction and professional skills are critical for rural populations to escape poverty (61). We found that among rural populations, individuals with higher levels of education have a lower risk of falling into poverty when they experience a health shock. This may be because education, as an important component of human capital, has a significant impact on the poverty alleviation of individuals and families. Previous studies have shown that the medical expenditure caused by health shocks will have a “crowding out effect” on human capital investment, which hinders the accumulation of household human capital (27). Low human capital stock is the most important factor that leads to poor people’s weak ability to get out of poverty, unstable income and unsustainable livelihood (62, 63). But better-educated individuals, who generally have more employment opportunities and higher incomes, are more resilient to risk shocks, reducing their likelihood of falling into poverty. Therefore, from the perspective of human capital, education is an effective way for rural families to get rid of poverty and get rich.

Previous research has shown that health shocks can lead to an increased risk of families falling into poverty (64). For rural residents in particular, limitations in professional skills and education levels prevent them from earning an income through stable employment. Through the mediation effect test, this paper finds that the health shock has a negative impact on the agricultural production income and wage income of rural households. Considering the fact that the income of China’s rural residents mainly depends on agricultural or non-agricultural employment, the decline in agricultural production income and wage income will inevitably lead to a decrease in total household income. Although some studies confirm that health shocks do not reduce the likelihood of family members participating in the labor production (65). Due to economic pressures, they often choose to continue working with their illness. In the long run, however, the decline in labor quality resulting from health shocks is detrimental to the accumulation of household wealth, thereby triggering the risk of poverty.

However, it should be noted that the research methods and approaches employed in this paper still require further refinement. First, in the context of rural China, health shocks are often one of the key risks that lead families into poverty. Therefore, the VEP method still has significant indicative significance for the vulnerability to health shocks. However, the vulnerable families identified by VEP may face multiple risks (including structural factors, family risk preferences, etc.), and policy interventions need to be made based on a comprehensive judgment and support, taking into account other information. Second, due to the availability of village-level data, although this study has mitigated the risk of omitted variable bias through instrumental variables and sensitivity analysis, it is still difficult to completely eliminate the interference from unobserved confounding factors at the village level. In the future, combining machine learning causal forests or field experiment designs could help us more deeply identify the causal path of the transmission of health poverty. Third, although our mediation analysis has confirmed the crucial role of income loss in affecting poverty vulnerability through health shocks, data limitations have prevented the examination of labor supply adjustments (such as reduction in working hours) or objective functional limitations (such as limitations in Activities of Daily Living). Future research can utilize the datasets of labor time surveys and clinical health assessments to further validate these approaches.

## 6 Conclusion and policy implications

Based on the data from the China Family Panel Studies (CFPS) from 2012 to 2020, this paper empirically analyzed the impact of health shocks on the poverty vulnerability of rural households. The main conclusions are as follows: First, the health shock significantly exacerbated the poverty vulnerability of rural families. Second, the impact of the health shock on poverty vulnerability varies by region and education level. Specifically, a higher level of education significantly mitigated the effect of the health shock on poverty vulnerability, but the mitigating effect was stronger under the absolute poverty standard. Compared to the eastern region, the exacerbating effect of the health shock on poverty vulnerability was more severe in the central and western regions, showing a gradient difference of “eastern region < central region < western region,” and the exacerbating effect under the absolute poverty standard was more obvious. Third, the health shock exacerbates the poverty vulnerability of families by reducing their agricultural production income and wage income. This study is helpful to prevent and cope with the risk of rural families returning to poverty, and explore the establishment of a long-term mechanism for healthy poverty alleviation. Based on the conclusions, we propose the following policy implications:

(1) Improve the medical security system and reduce the direct economic burden of health shocks. In the central and western regions, the impact of health shocks on the vulnerability to poverty is the most significant, which is closely related to the relatively low level of medical security in these areas. Therefore, policies should prioritize expanding the coverage of critical illness insurance in regions with scarce medical resources and increasing the reimbursement rate, especially for the long-term treatment costs of chronic and major diseases. It is suggested that chronic diseases (such as diabetes and hypertension) and long-term rehabilitation treatments in rural areas of the central and western regions should be covered by the insurance, and the reimbursement rate should be increased; for high-incidence diseases (such as cancer, cardiovascular and cerebrovascular diseases), free early screening should be provided to reduce the economic burden of late-stage treatment. At the same time, efforts can be made to establish a “health shock early warning mechanism.” When family medical expenses exceed 30% of the annual income, automatic triggers will occur for temporary medical assistance, fee reduction or installment payment policies, preventing families from falling into debt crises due to medical expenses. Furthermore, the government should enhance the capacity-building of primary-level medical services, improve the medical treatment capabilities of township health centers and village clinics, and reduce the risk of poverty caused by illness.
(2) Strengthening investment in human capital for education, and enhancing the ability of families to withstand health risks. The research reveals that families with higher educational levels demonstrate greater resilience when facing health shocks, indicating that education not only enhances income capabilities but also strengthens the awareness of risk management. Therefore, policies should further strengthen educational support in rural areas, particularly in improving the consolidation rate of compulsory education in the central and western regions. Specific measures include: Implementing the “Dropout Prevention Guarantee Program” during the junior high school stage, providing financial assistance and boarding subsidies to children from families affected by illness, to ensure they complete 9 years of compulsory education. During the high school and vocational education stages, the government can expand the coverage of the “Rainbow Project,” providing vocational skills training for low-income families to enhance their non-agricultural employment capabilities. For the adult workforce, promote “health literacy education,” and through community training, enhance the ability to prevent diseases and manage health, thereby reducing the probability of health shocks.
(3) Establish a diversified income guarantee mechanism to alleviate the indirect economic impact of health shocks. The health shock mainly exacerbates poverty vulnerability by reducing agricultural income and wage income. Therefore, policies need to establish a multi-level income support system. For families engaged in agricultural production, the government can promote the leasing service of small agricultural machinery. The government will subsidize 50% of the rent, thereby reducing the reliance on physical labor; establish “mutual aid agricultural cooperatives,” allowing sick families to invest their land and having healthy laborers cultivate the land on their behalf. For non-agricultural employment households, more support for flexible employment should be provided. For instance, through “work-for-relief” programs, temporary employment opportunities can be offered; or in collaboration with enterprises, “family-friendly” job positions can be developed, allowing caregivers of sick family members to engage in flexible employment. Furthermore, it is possible to explore the establishment of a “health shock emergency fund,” which would be jointly funded by local governments, charitable organizations and village-level cooperatives. This fund would provide small interest-free loans or direct cash transfers to affected families to help them overcome the difficult period of sudden income decline. These measures should be combined with the existing rural revitalization policies to form a sustainable anti-poverty guarantee system.

## Data Availability

The original contributions presented in the study are included in the article/supplementary material, further inquiries can be directed to the corresponding author.
